# Non-invasive imaging through strongly scattering media based on speckle pattern estimation and deconvolution

**DOI:** 10.1038/s41598-018-27467-1

**Published:** 2018-06-14

**Authors:** Zhouping Wang, Xin Jin, Qionghai Dai

**Affiliations:** Graduate School at Shenzhen, Tsinghua University, Shenzhen, 518055 China

## Abstract

Imaging through scattering media is still a formidable challenge with widespread applications ranging from biomedical imaging to remote sensing. Recent research progresses provide several feasible solutions, which are hampered by limited complexity of targets, invasiveness of data collection process and lack of robustness for reconstruction. In this paper, we show that the complex to-be-observed targets can be non-invasively reconstructed with fine details. Training targets, which can be directly reconstructed by speckle correlation and phase retrieval, are utilized as the input of the proposed speckle pattern estimation model, in which speckle modeling and constrained least square optimization are applied to estimate the distribution of the speckle pattern. Reconstructions for to-be-observed targets are realized by deconvoluting the estimated speckle pattern from the acquired integrated intensity matrices (IIMs). The qualities of reconstructed results are ensured by the stable statistical property and memory effect of laser speckle patterns. Experimental results show that the proposed method can reconstruct complex targets in high quality and the reconstruction performance is robust even much less data are acquired.

## Introduction

Light scattering, caused by inhomogeneous refractive index presented in the transmission media, limits the resolution and the signal-to-noise ratio of optical imaging^1^. Random refraction in the scattering media encodes the spatial information of imaging targets into scrambled images and causes the speckle noise. Ballistic light based methods, such as optical coherent tomography^2–4^ and two-photon microscopy^5,6^, extend the imaging depth by extracting photons that have not been scattered. Adaptive optics^7^ can correct the low-order deformations using deformable mirrors. However, when the scattering media diffuses nearly all the light going through it^1,8^, the approaches mentioned above will be out of action. Target localization inside the scattering media can be realized by diffuse optical tomography^9,10^, whose imaging resolution is limited by scattering depth. Some recent works^11–20^ based on memory effect^21–23^ provide novel imaging solutions, including wavefront-shaping^11–14^, speckle correlation^15–17^, bispectrum analysis^18^ and deconvolution^19,20^, for strongly scattering situations. Controlled wavefront-shaping allows imaging and focusing through scattering media by modulating the degrees of freedom in the scattered waves^24–37^. Furthermore, up-to-date ‘guidestar’ mechanisms^38–42^ provide non-invasive feedback for intra-tissue focusing. Nevertheless, the imaging resolution is limited by the size of illumination speckle grain, and the field of view (FoV) for high light-transmission enhancement is limited in the vicinity of the focusing spot. Speckle correlation based approaches, which include speckle scanning^15,16^ and single-shot^17^ techniques, collect the convolution of the target and the point-spread speckle pattern^43,44^. The autocorrelation computation and Fourier transform are utilized to get the amplitude spectrum of targets, and phase-retrieval algorithms^45,46^ to realize reconstruction. However, the methods require high-resolution angular sampling to weaken the effect caused by speckle noise. Also, the reconstruction quality is limited by the size of the illumination speckle grain, and the stability of phase-retrieval algorithm is restricted by the structural complexity of imaging targets. Bispectrum analysis based method^18^, which can deterministically and unambiguously extract the Fourier phase of the target from scattered light pattern, also suffers from the problems caused by the stochastic perturbation of speckle pattern. Deconvolution based approaches can realize imaging with resolution beyond the diffraction limitation, while the main problem is how to acquire the point-spread speckle pattern. Point-spread-function (PSF) capturing based deconvolution method^19,20^ needs to put a point source behind the scattering media during the estimation process, whose invasiveness impedes the practicability for many real applications.

To non-invasively image complex targets through strongly scattering media with resolution beyond the limitation of illumination speckle grain lighted on the targets, a preliminary speckle pattern estimation and deconvolution method was proposed in our previous work^47^. The method is extended in this paper by adding cross-correlation matching method for restoring orientations and positions, deriving theoretical model for the speckle pattern estimation, analyzing the solution space and data reduction and comparing experimental results for imaging targets with different sizes and structural complexities. Imaging system based on speckle correlation^15^ is utilized to collect IIMs of the to-be-observed targets. Exploiting the frequency-bounded characteristics of the speckle pattern^48^ and the convolutional relationship between the imaging target and the speckle pattern, speckle estimation based on the constrained least square optimization and speckle modeling is proposed to estimate the speckle pattern distribution. IIMs are aligned by the orientational and positional information recovered from the cross-correlation matching method. The complexity in solving the speckle pattern estimation model and the quality of estimation using the training targets in different shapes with different combinations are discussed in detail. Deconvolution is performed to reconstruct targets using their corresponding IIMs and the estimated speckle pattern. Reconstruction using much less acquired IIM data is also analyzed to demonstrate the feasibility in reducing the data collection complexity. Compared with speckle correlation^15–17^ and bispectrum analysis^18^ based approaches, in which the stochastic perturbations in point-spread speckle pattern are considered as statistic noises, our method directly estimates the speckle pattern and reconstruct the target by deconvolution algorithm. The demand of high-resolution angular sampling is relieved and it can recover high-quality details of complex targets with directional information. Experimental results and discussions on real-captured data are provided to verify the effectiveness of the proposed method. Compared with the existing approaches, the proposed method provides much higher reconstruction quality to much more complex to-be-observed targets with much larger FoV.

## Principles and Methods

### Experimental setup and denotations

**Figure 1:**
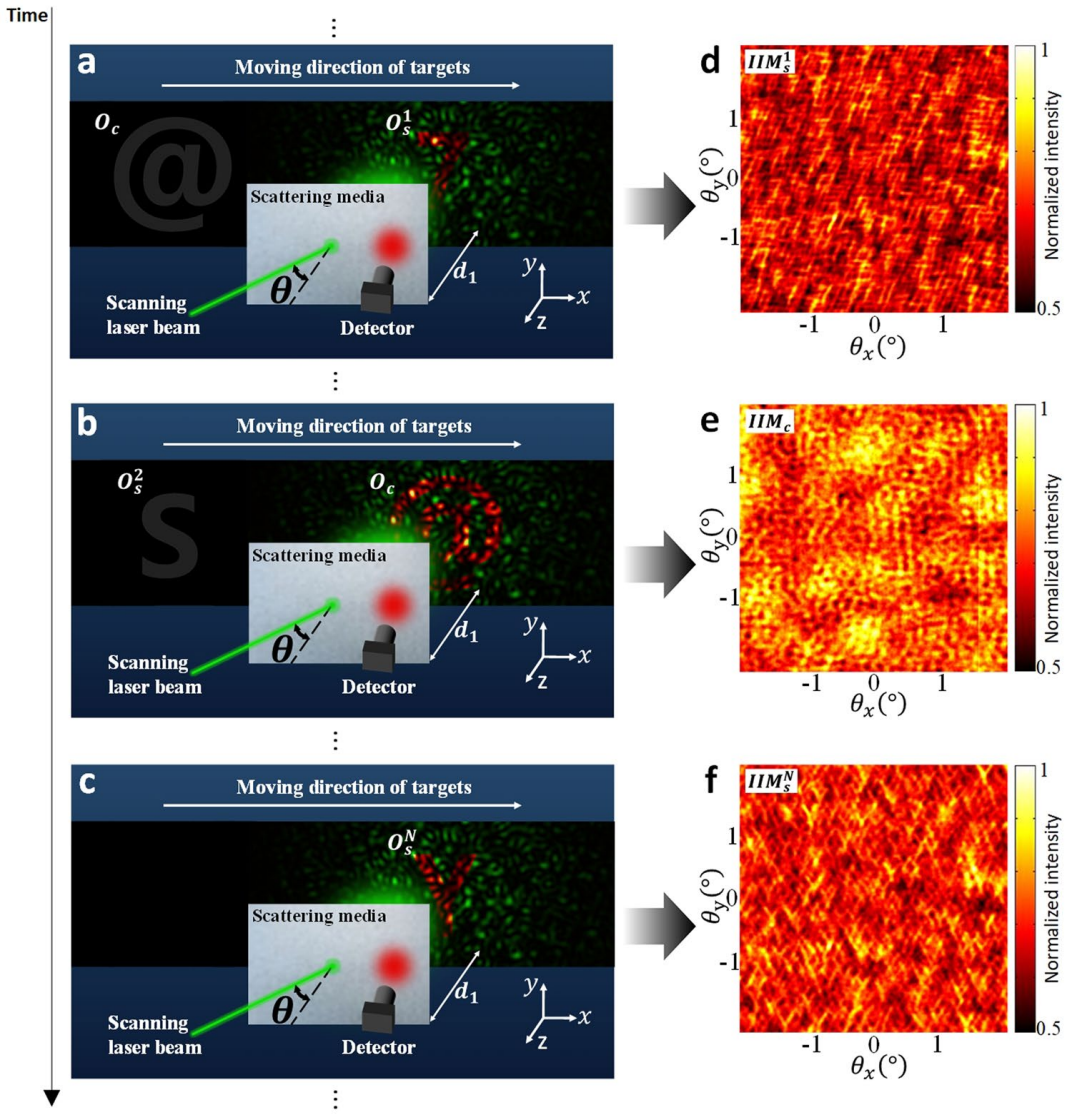
Schematic diagram of data collection. (**a**–**c**) ${O}_{s}^{i}$ and ${O}_{c}$ are training target and to-be-observed target, respectively. (**d**–**f**) are the IIMs, corresponding to (**a**–**c**), respectively, collected by the detector as the laser beam lights through the scattering media with the incident angle $\theta =({\theta }_{x},{\theta }_{y})$.

The schematic diagram of data collection process is illustrated in Fig. 1. In the enclosed environment surrounded by the scattering media, several imaging targets with different structural complexities may appear in the imaging region. The targets that can be reconstructed by phase-retrieval algorithm, namely training targets, are denoted by ${O}_{s}^{i}(i=1,2,\cdots ,N)$. And the to-be-observed targets are denoted by *O*_*c*_. Imaging system based on raster scanning and speckle correlation, which was first proposed by Bertolotti *et al*.^15^, is utilized to collect the IIMs for the imaging targets. When imaging target is in the common imaging region behind the scattering media, the laser beam with incident angle of $({\theta }_{x},{\theta }_{y})$ will generate speckle pattern lighting on the target through the scattering media. In case of the existence of memory effect^21–23^, the point-spread speckle pattern can be approximately expressed by $S(x-{\theta }_{x}{d}_{1},y-{\theta }_{y}{d}_{1})$, where *d*_1_ is the distance between the imaging target and the scattering media. The intensity of the speckle pattern transmitted and diffused by the scattering media, which encodes the information of the imaging target, is recorded by the photon detector. Imaging target can be generally denoted by $O(x-{r}^{(1)},y-{r}^{(2)})$, where (*x*, *y*) is the coordinate on the object plane and $({r}^{(1)},{r}^{(2)})$ is its spatial position relative to the center of the FoV. The recorded integrated intensity corresponding to a specific incident angle of laser beam, $({\theta }_{x},{\theta }_{y})$, can be expressed as

1 $$\begin{array}{rcl}IIM({\theta }_{x},{\theta }_{y}) & = & \int {\int }_{-\infty }^{\infty }O(x-{r}^{(1)},y-{r}^{(2)})\,S(x-{\theta }_{x}{d}_{1},y-{\theta }_{y}{d}_{1})dxdy\\  & = & [O\ast S]({d}_{1}{\theta }_{x}-{r}^{(1)},{d}_{1}{\theta }_{y}-{r}^{(2)})\end{array},$$

where * denotes the convolution operator and IIM is the integrated intensity matrix. The pixel intensity of IIM corresponds to the total intensity of the image captured at a laser incident angle. The sample IIMs collected for ${O}_{s}^{1}$, ${O}_{c}$ and ${O}_{s}^{N}$ in Fig. 1(a–c) are shown in Fig. 1(d–f), respectively.

### Speckle pattern estimation

From Eq. (1), it can be observed that once the point-spread speckle pattern that scans the imaging region is known, theoretically, the target can be reconstructed by deconvoluting *S* from IIM by^47^

2 $$O={ {\mathcal F} }^{-1}\{\frac{ {\mathcal F} (IIM)}{ {\mathcal F} (S)}\},$$

where ${\mathcal F}$ and ${ {\mathcal F} }^{-1}$ represent Fourier transformation and inverse Fourier transformation, respectively. However, in general, the point-spread speckle pattern is unknown, which can only be estimated.

To estimate the speckle pattern, the convolutional relationships among the speckle pattern, $S$, training targets, ${O}_{s}^{i}(i=1,2,\cdots ,N)$, and their IIMs, $II{M}_{s}^{i}(i=1,2,\cdots ,N)$, are exploited. Denoting the phase-retrieved results of training targets as ${\bar{O}}_{s}^{i}(i=1,2,\cdots ,N)$, the convolutional relationship defined in Eq. (1) can be converted to be

3 $$II{M}_{s}^{i}={\bar{O}}_{s}^{i}\ast S+{e}_{i},i=1,2,\cdots ,N,$$

where ${e}_{i}$ represents the error caused by imaging noise and phase-retrieval algorithm. ${\bar{O}}_{s}^{i}\in {R}^{{M}_{x}\times {M}_{y}}$, $II{M}_{s}^{i}\in {R}^{{K}_{x}\times {K}_{y}}$, $S\in {R}^{({M}_{x}+{K}_{x}-1)\times ({M}_{y}+{K}_{y}-1)}$ and ${e}_{i}\in {R}^{{K}_{x}\times {K}_{y}}$, where ${M}_{x}\times {M}_{y}$ is the dimension of the imaging target and ${K}_{x}\times {K}_{y}$ is the number of sampling points in $II{M}_{s}^{i}$. It is a linear system with $({M}_{x}+{K}_{x}-1)({M}_{y}+{K}_{y}-1)$ variables and $N{K}_{x}{K}_{y}$ equations. Solving *s* in Eq. (3) is an underdetermined problem and ${e}_{i}$ is also unknown. Considering the intensity distribution of speckle pattern is always positive and it presents frequency-bounded characteristics inherited from Fourier transformation of the laser beam wavefront^48^, the prior of speckle pattern distribution is introduced to build a constrained least square model for speckle estimation as^47^

4 $$\begin{array}{rcl}\ddot{S} & = & \text{arg}\,\mathop{\min }\limits_{S}\sum _{i=1}^{N}{\Vert {e}_{i}\Vert }_{F}^{2}=\text{arg}\,\mathop{\min }\limits_{S}\,\sum _{i=1}^{N}{\Vert II{M}_{s}^{i}-{\overline{O}}_{s}^{i}\ast S\Vert }_{F}^{2}\\  &  & {s}.\,{t}.\,0\le {S}_{m,n}\le 1,-\,{\rm{\Delta }}\le {S}_{m,n+1}-{S}_{m,n}\le {\rm{\Delta }},-\,{\rm{\Delta }}\le {S}_{m+1,n}-{S}_{m,n}\le {\rm{\Delta }}\end{array},$$

where ${\rm{\Delta }}$ constrains the variation between the intensity of two adjacent points in the speckle pattern. ${\rm{\Delta }}$ is inversely proportional to the size of the illumination speckle grain, and the speckle grain size is proportional to *d*_1_ and wavelength of the laser while inversely proportional to the diameter of laser beam^48^. The speckle estimation model in Eq. (4) is a convex problem^49^, which can be solved by the solvers of convex optimization problem (CVX^50,51^ is used in our experiments).

### Phase retrieval and cross-correlation matching for reconstruction of training targets

The phase-retrieval reconstruction of training target, denoted by ${\overline{O}}_{s}^{i}$, is used as the input of Eq. (4). To reconstruct ${\overline{O}}_{s}^{i}$, the sharply peaked property of the average autocorrelation of speckle pattern^52^ is exploited first. Through autocorrelation of IIM and plugging Eq. (1) into it, the Fourier magnitude spectrum of ${O}_{s}^{i}$ is given by^15^

5 $$\sqrt{ {\mathcal F} (II{M}_{s}^{i}\ast II{M}_{s}^{i})}=\sqrt{ {\mathcal F} [({O}_{s}^{i}\ast {O}_{s}^{i})\ast (S\ast S)]}\approx \sqrt{ {\mathcal F} ({O}_{s}^{i}\ast {O}_{s}^{i})}=| {\mathcal F} ({O}_{s}^{i})|,$$

where * denotes the correlation operator. Then, phase-retrieval algorithm, Hybrid Input-Output and Error-Reduction^15,45,46^, is applied to recover ${\overline{O}}_{s}^{i}$ via iteratively updating the phases with fixed magnitude spectrum.

After phase retrieval, the orientational and positional information of ${\overline{O}}_{s}^{i}$ is lost. Also, the training targets may not be spatially aligned in the imaging region. It leads to the spatial disconformity between the captured IIMs with the convolutions of ${\overline{O}}_{s}^{i}$ and *S*. Directly putting ${\overline{O}}_{s}^{i}$ and the captured $II{M}_{s}^{i}$ into Eq. (4) will result in mismatches and cannot estimate the local spatial distributions of speckle pattern accurately. Thus, cross-correlation matching is proposed to correct the orientations of ${\overline{O}}_{s}^{i}$ and to align $II{M}_{s}^{i}$ in Eq. (4).

According to Eq. (1), the IIM of ${O}_{s}^{i}$ can be expressed as

6 $$II{M}_{s}^{i}({\theta }_{x},{\theta }_{y})=[{O}_{s}^{i}\ast S]({d}_{1}{\theta }_{x}-{r}_{i}^{(1)},{d}_{1}{\theta }_{y}-{r}_{i}^{(2)}),$$

where $({r}_{i}^{(1)},{r}_{i}^{(2)})$ is the spatial position of ${O}_{s}^{i}$ relative to the center of FoV. The autocorrelation of speckle pattern is a sharply peaked function, which can be expressed by^48^

7 $$[S\ast S](x,y)=B\delta (x,y)+C$$

where $\delta$ denotes impulse function; *B* and *C* are the constants depending on the size of speckle grain. Applying cross-correlation to the IIMs of two training targets ${O}_{s}^{i}$ and ${O}_{s}^{j}$, we have

8 $$\begin{array}{rcl}[II{M}_{s}^{i}\ast II{M}_{s}^{j}]({\theta }_{x},{\theta }_{y}) & = & \frac{B}{{d}_{1}^{2}}[{O}_{s}^{i}\ast {O}_{s}^{j}]({d}_{1}{\theta }_{x}+{r}_{j}^{(1)}-{r}_{i}^{(1)},{d}_{1}{\theta }_{y}+{r}_{j}^{(2)}-{r}_{i}^{(2)})+\frac{C}{{d}_{1}^{2}}\int {\int }_{-\infty }^{\infty }[{O}_{s}^{i}\ast {O}_{s}^{j}](x,y)dxdy\\  & = & \frac{B}{{d}_{1}^{2}}[{O}_{s}^{i}\ast {O}_{s}^{j}]({d}_{1}{\theta }_{x}+{r}_{j}^{(1)}-{r}_{i}^{(1)},{d}_{1}{\theta }_{y}+{r}_{j}^{(2)}-{r}_{i}^{(2)})+D.\end{array}$$

For phase-retrieved result of ${O}_{s}^{i}$, i.e. ${\overline{O}}_{s}^{i}$, there are two possible orientations: the orientation of ${\overline{O}}_{s}^{i}$ is the same with that of ${O}_{s}^{i}$ as

9 $${\overline{O}}_{s}^{i}({d}_{1}{\theta }_{x},{d}_{1}{\theta }_{y})\approx {O}_{s}^{i}({d}_{1}{\theta }_{x},{d}_{1}{\theta }_{y})$$

and ${\overline{O}}_{s}^{i}$ is the flipped of ${O}_{s}^{i}$ as

10 $${\overline{O}}_{s}^{i}({d}_{1}{\theta }_{x},{d}_{1}{\theta }_{y})\approx {O}_{s}^{i}(\,-\,{d}_{1}{\theta }_{x},-\,{d}_{1}{\theta }_{y}).$$

Thus, by comparing the four possible orientation combinations of ${\overline{O}}_{s}^{i}$ and ${\overline{O}}_{s}^{j}$, the orientation which maximizes the correlation between the correlation of rotated phase-retrieved results and the correlation of IIMs is achieved by

11 $$\begin{array}{rcl}{\varepsilon }_{i},{\varepsilon }_{j} & = & {\rm{\arg }}\mathop{{\rm{\max }}}\limits_{{\varepsilon }_{i},{\varepsilon }_{j}=0;1}\,{\rm{\max }}\,\{[{\overline{O}}_{s}^{i}({(-1)}^{{\varepsilon }_{i}}{d}_{1}{\theta }_{x},{(-1)}^{{\varepsilon }_{i}}{d}_{1}{\theta }_{y})\ast {\overline{O}}_{s}^{j}({(-1)}^{{\varepsilon }_{j}}{d}_{1}{\theta }_{x},{(-1)}^{{\varepsilon }_{j}}{d}_{1}{\theta }_{y})]\\  &  & \ast \,\{[II{M}_{s}^{i}\ast II{M}_{s}^{j}]({\theta }_{x},{\theta }_{y})-D\}\}.\end{array}$$

As a result, the reconstructed training targets with correct orientations are obtained by

12 $${\ddot{O}}_{s}^{i}({d}_{1}{\theta }_{x},{d}_{1}{\theta }_{y})={\overline{O}}_{s}^{i}({(-1)}^{{\varepsilon }_{i}}{d}_{1}{\theta }_{x},{(-1)}^{{\varepsilon }_{i}}{d}_{1}{\theta }_{y}).$$

Since ${\ddot{O}}_{s}^{i}$ is an approximation of ${O}_{s}^{i}$ with some reconstruction errors, the spatial relative offsets between ${O}_{s}^{i}$ and ${O}_{s}^{j}$ can be estimated by

13 $$({r}_{j}^{(1)}-{r}_{i}^{(1)},{r}_{j}^{(2)}-{r}_{i}^{(2)})=\text{arg}\mathop{\max }\limits_{{\rm{\Delta }}{r}_{ij}^{(1)},{\rm{\Delta }}{r}_{ij}^{(2)}}\{[{\ddot{O}}_{s}^{i}\ast {\ddot{O}}_{s}^{j}]\ast \{[II{M}_{s}^{i}\ast II{M}_{s}^{j}]-D\}\}(\frac{{\rm{\Delta }}{r}_{ij}^{(1)}}{{d}_{1}},\frac{{\rm{\Delta }}{r}_{ij}^{(2)}}{{d}_{1}}).$$

Then, $II{M}_{s}^{i}(i=2,\cdots ,N)$ is aligned to $II{M}_{s}^{1}$ by

14 $$I\dot{I}{\dot{M}}_{s}^{i}({\theta }_{x},{\theta }_{y})=II{M}_{s}^{i}({d}_{1}{\theta }_{x}-({r}_{1}^{(1)}-{r}_{i}^{(1)}),{d}_{1}{\theta }_{y}-({r}_{1}^{(2)}-{r}_{i}^{(2)}))=[{O}_{s}^{i}\ast S]({d}_{1}{\theta }_{x}-{r}_{1}^{(1)},{d}_{1}{\theta }_{y}-{r}_{1}^{(2)}).$$

Finally, the speckle estimation model in Eq. (4) is updated to be

15 $$\begin{array}{rcl}\ddot{S} & = & \text{arg}\,\mathop{\min }\limits_{S}\sum _{i=1}^{N}\parallel {e}_{i}{\parallel }_{F}^{2}=\text{arg}\mathop{\min }\limits_{S}\sum _{i=1}^{N}\parallel I\dot{I}{\dot{M}}_{s}^{i}-{\ddot{O}}_{s}^{i}\ast S{\parallel }_{F}^{2}\\  &  & {s}.\,{t}.\,0\le {S}_{m,n}\le 1,-\,{\rm{\Delta }}\le {S}_{m,n+1}-{S}_{m,n}\le {\rm{\Delta }},-\,{\rm{\Delta }}\le {S}_{m+1,n}-{S}_{m,n}\le {\rm{\Delta }},\end{array}$$

which uses aligned IIMs and orientation-corrected phase-retrieved results to provide a theoretical optimization solution matching the original distribution of *S*.

## Model Analysis

### Training targets analysis

**Figure 2:**
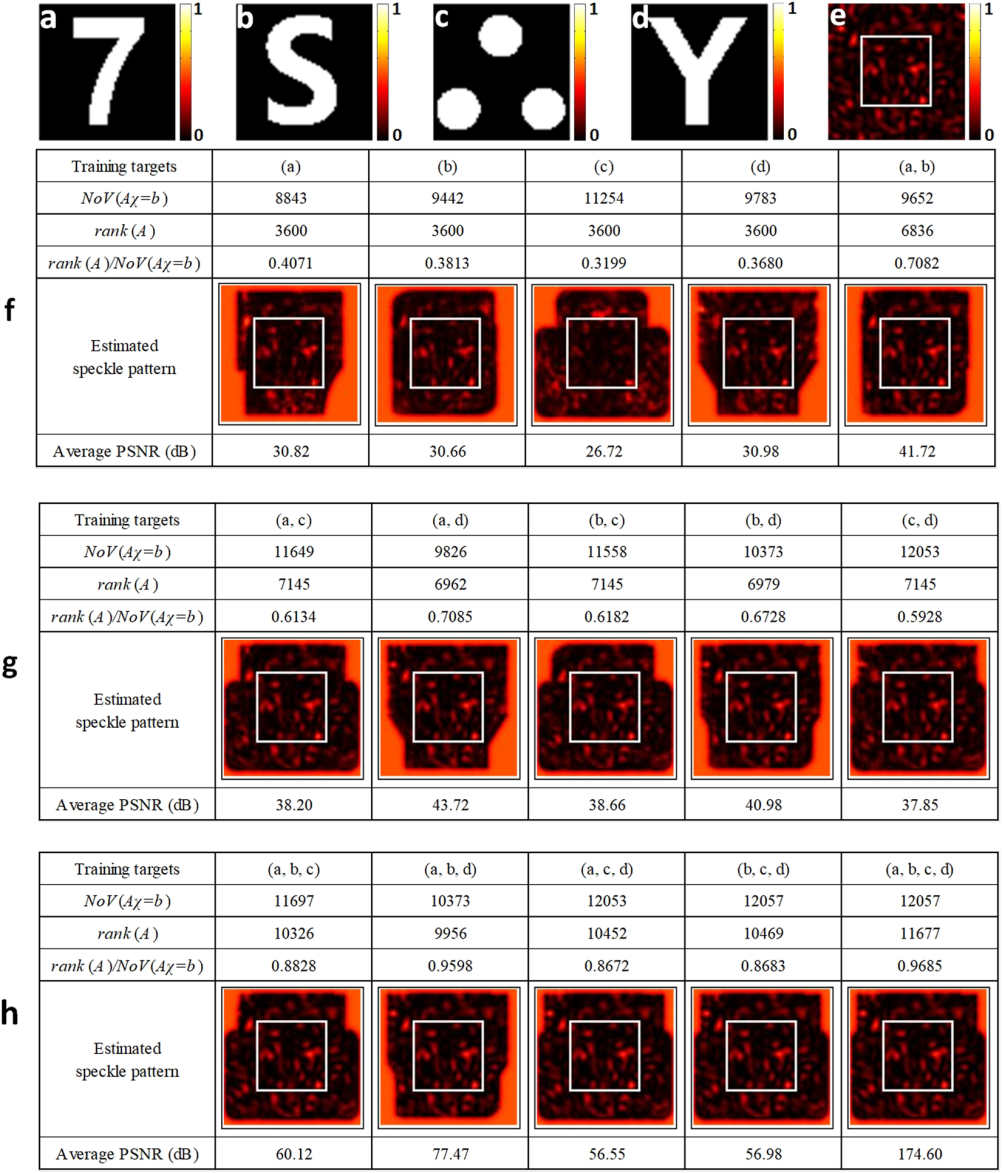
Simulated experiment for comparing the speckle pattern estimation performance. (**a**–**d**) Training targets in size of 60 × 60 $({M}_{x},{M}_{y}=60)$; (**e**) A sample of speckle pattern ground truth in size of 119 × 119 $({M}_{x}+{K}_{x}-1=119,{M}_{y}+{K}_{y}-1=119)$ used for raster scanning the training targets, where ${K}_{x}\times {K}_{y}=60\times 60$ is the number of points for raster scanning, i.e. the size of IIMs. (**f**–**h**) Speckle pattern estimation results with different combinations of training targets.

The speckle estimation performance of Eq. (15) is decided by the shape, size and the number of training targets. The increase in the number of training targets, the larger shape differences among training targets and the size reduction of each target will result in better estimation performance. However, the estimation qualities of entries in matrix *S* are not the same. The objective function of the estimation model, Eq. (15), is the least square solution of linear equations,

16 $$I\dot{I}{\dot{M}}_{s}^{i}={\ddot{O}}_{s}^{i}\ast S,i=1,2,\cdots ,N,$$

in which the entries of *S* in the central are included in more independent equations than those in the margins. Thus, the central entries have smaller solution spaces and higher probabilities to be solved with lower errors. Eq. (16) can also be discretely formed by

17 $$A\chi =b,$$

where

18 $$\begin{array}{cc}\chi [({M}_{y}+{K}_{y}-1)(m-1)+n]=S(m{\rm{\Delta }}{\theta }_{x}{d}_{1},n{\rm{\Delta }}{\theta }_{y}{d}_{1}), & 1\le m\le {M}_{x}+{K}_{x}-1,1\le n\le {M}_{y}+{K}_{y}-1\end{array},$$

19 $$\begin{array}{c}A[{K}_{x}{K}_{y}(i-1)+{K}_{y}(m-1)+n,({M}_{y}+{K}_{y}-1)(m+p-2)+(n+q-1)]={\ddot{O}}_{s}^{i}(p{\rm{\Delta }}{\theta }_{x}{d}_{1},q{\rm{\Delta }}{\theta }_{y}{d}_{1}),\\ \,\,\,\,\,\,\,\,\,\,\,\,\,\,\,\,\,\,\,1\le m\le {K}_{x},1\le n\le {K}_{y},1\le p\le {M}_{x},1\le q\le {M}_{y},\end{array}$$

20 $$b[{K}_{x}{K}_{y}(i-1)+{K}_{y}(m-1)+n]=I\dot{I}{\dot{M}}_{s}^{i}(m{\rm{\Delta }}{\theta }_{x},n{\rm{\Delta }}{\theta }_{y}),\,1\le m\le {K}_{x},1\le n\le {K}_{y},$$

in which $({\rm{\Delta }}{\theta }_{x},{\rm{\Delta }}{\theta }_{y})$ is the sampling interval of IIMs and the values of undefined elements in matrix *A* are zeros. The increase in the number of training targets with different shapes leads to the increase in the number of variables, denoted by $NoV(A\chi =b)$, and the increase in the rank of *A*, denoted by $rank(A)$. $NoV(A\chi =b)$ equals to the number of nonzero columns in matrix *A*. Simulations in Fig. 2 compare the speckle pattern estimation performance using the training targets in different shapes with different combinations. Four training targets in size of 60 × 60 $({M}_{x},{M}_{y}=60)$, as shown in Fig. 2(a–d), are selected. Speckle patterns at object plane are generated by simulating the wave propagation of random phased laser beam with Fresnel diffraction equation^43^. Raster scanning process (corresponds to changing the incident angle of laser beam with the memory effect^21–23^) is simulated to acquire the IIMs in size of 60 × 60 $({K}_{x},{K}_{y}=60)$. To get rid of the reconstruction error caused by phase-retrieval algorithm, original training targets are utilized to estimate the speckle patterns using the proposed model in Eq. (15). Cropping the estimated speckle patterns in size of 60 × 60 in the center, i.e. the region lined in white, the peak signal to noise ratio (PSNR) is calculated using the simulated ground truth, as an example shown in Fig. 2(e), as the reference. 100 simulated speckle patterns are tested to average the PSNR for a robust result. As we can see from the results, the estimation quality is positively related to the number of the training targets used. For example, the estimation quality using two training targets is much higher than only using one training target, while much lower than using three or four training targets. Also, it can be found that the estimation quality is mainly determined by the rank-variable ratio, i.e. $rank(A)/NoV(A\chi =b)$. When the number of training targets is increased, the increase in the number of independent equations, i.e. *rank*(*A*), is larger than that in the number of variables, i.e. $NoV(A\chi =b)$. And it results in the increase of rank-variable ratio. Since the higher rank-variable ratio corresponds to the less underdetermined variables, the estimation quality can be improved obviously. The variation in the shape of the training targets also results in the change of the rank-variable ratio. If the targets with higher structural complexities can be reconstructed well by phase retrieval, the bigger structural differences among the training targets result in the higher *rank*(*A*), which benefits the speckle pattern estimation.

### Reconstruction with less data

After getting the estimated speckle pattern $\ddot{S}$ from Eq. (15), deconvolution can be executed for the acquired IIM to reconstruct an imaging target with much higher accuracy and robustness than the phase-retrieval methods. It is also observed that we can further reduce the data acquisition complexity for acceptable reconstruction quality. Considering that the collection of IIM by raster scanning and memory effect as proposed by Bertolotti *et al*.^15^ is a discrete sampling process, the discrete representation of IIM for a to-be-observed target can be expressed by updating Eq. (1) to

21 $$\begin{array}{rcl}{\overline{IIM}}_{c}(m,n) & = & II{M}_{c}(m{\rm{\Delta }}{\theta }_{x},n{\rm{\Delta }}{\theta }_{y})\\  & = & {\rm{\Delta }}{\theta }_{x}{\rm{\Delta }}{\theta }_{y}{d}_{1}^{2}\int {\int }_{-\infty }^{\infty }{O}_{c}(u{\rm{\Delta }}{\theta }_{x}{d}_{1}-{r}_{c}^{(1)},v{\rm{\Delta }}{\theta }_{y}{d}_{1}-{r}_{c}^{(2)})S(u{\rm{\Delta }}{\theta }_{x}{d}_{1}\\  &  & -m{\rm{\Delta }}{\theta }_{x}{d}_{1},v{\rm{\Delta }}{\theta }_{y}{d}_{1}-n{\rm{\Delta }}{\theta }_{y}{d}_{1})dudv\\  & \approx  & {\rm{\Delta }}{\theta }_{x}{\rm{\Delta }}{\theta }_{y}{d}_{1}^{2}\sum _{q=-\infty }^{\infty }\sum _{p=-\infty }^{\infty }{\overline{O}}_{c}(p,q)\overline{S}(p-m,q-n)\end{array},$$

where ${\overline{IIM}}_{c}$, ${\overline{O}}_{c}$ and $\overline{S}$ are the discrete sampling signals of $II{M}_{c}({\theta }_{x},{\theta }_{y})$, ${O}_{c}(x-{r}_{c}^{(1)},y-{r}_{c}^{(2)})$ and $S(x,y)$ with the sampling intervals as $({\rm{\Delta }}{\theta }_{x},{\rm{\Delta }}{\theta }_{y})$, $({\rm{\Delta }}{\theta }_{x}{d}_{1},{\rm{\Delta }}{\theta }_{y}{d}_{1})$ and $({\rm{\Delta }}{\theta }_{x}{d}_{1},{\rm{\Delta }}{\theta }_{y}{d}_{1})$, respectively; $({r}_{c}^{(1)},{r}_{c}^{(2)})$ is the position of the to-be-observed target.

As the sampling interval becomes to be $\{(H{\rm{\Delta }}{\theta }_{x},H{\rm{\Delta }}{\theta }_{y})|H\in {Z}^{+},H > 1\}$, corresponding to reducing the complexity in data acquisition by ${H}^{2}$ times, the collected IIM becomes to be

22 $$\begin{array}{rcl}{\overline{IIM}}_{c}(Hm,Hn) & = & II{M}_{c}(Hm{\rm{\Delta }}{\theta }_{x},Hn{\rm{\Delta }}{\theta }_{y})\\  & = & {H}^{2}{\rm{\Delta }}{\theta }_{x}{\rm{\Delta }}{\theta }_{y}{d}_{1}^{2}\int {\int }_{-\infty }^{\infty }{O}_{c}(Hu{\rm{\Delta }}{\theta }_{x}{d}_{1}-{r}_{c}^{(1)},Hv{\rm{\Delta }}{\theta }_{y}{d}_{1}-{r}_{c}^{(2)})S(Hu{\rm{\Delta }}{\theta }_{x}{d}_{1}\\  &  & -Hm{\rm{\Delta }}{\theta }_{x}{d}_{1},Hv{\rm{\Delta }}{\theta }_{y}{d}_{1}-Hn{\rm{\Delta }}{\theta }_{y}{d}_{1})dudv\\  & \approx  & {H}^{2}{\rm{\Delta }}{\theta }_{x}{\rm{\Delta }}{\theta }_{y}{d}_{1}^{2}\sum _{q=-\infty }^{\infty }\sum _{p=-\infty }^{\infty }{\overline{O}}_{c}(Hp,Hq)\overline{S}(Hp-Hm,Hq-Hn)\end{array},$$

Thus, using the down-sampled estimated speckle pattern, denoted by $\overline{\ddot{S}}$, it is still possible to reconstruct the imaging target in lower resolution by deconvoluting ${\overline{IIM}}_{c}$ with $\overline{\ddot{S}}$.

## Results and Discussion

In this section, the performance of the proposed speckle pattern estimation model is demonstrated by testing on a real imaging system, in which two Edmund Optics 120-grit ground-glass diffusers^15^ are used as the scattering media. In this system, speckle scanning and recovery method proposed by Bertolotti *et al*.^15^ is used. The laser beam is controlled by two galvanometric scanners in vertical and horizontal directions separately to generate different incident angles at the scattering media. The details of the imaging system can be found in the electronic supplementary material. To reduce the noises and artifacts, Richardson-Lucy deconvolution algorithm^53,54^ is applied for reconstruction.

**Figure 3:**
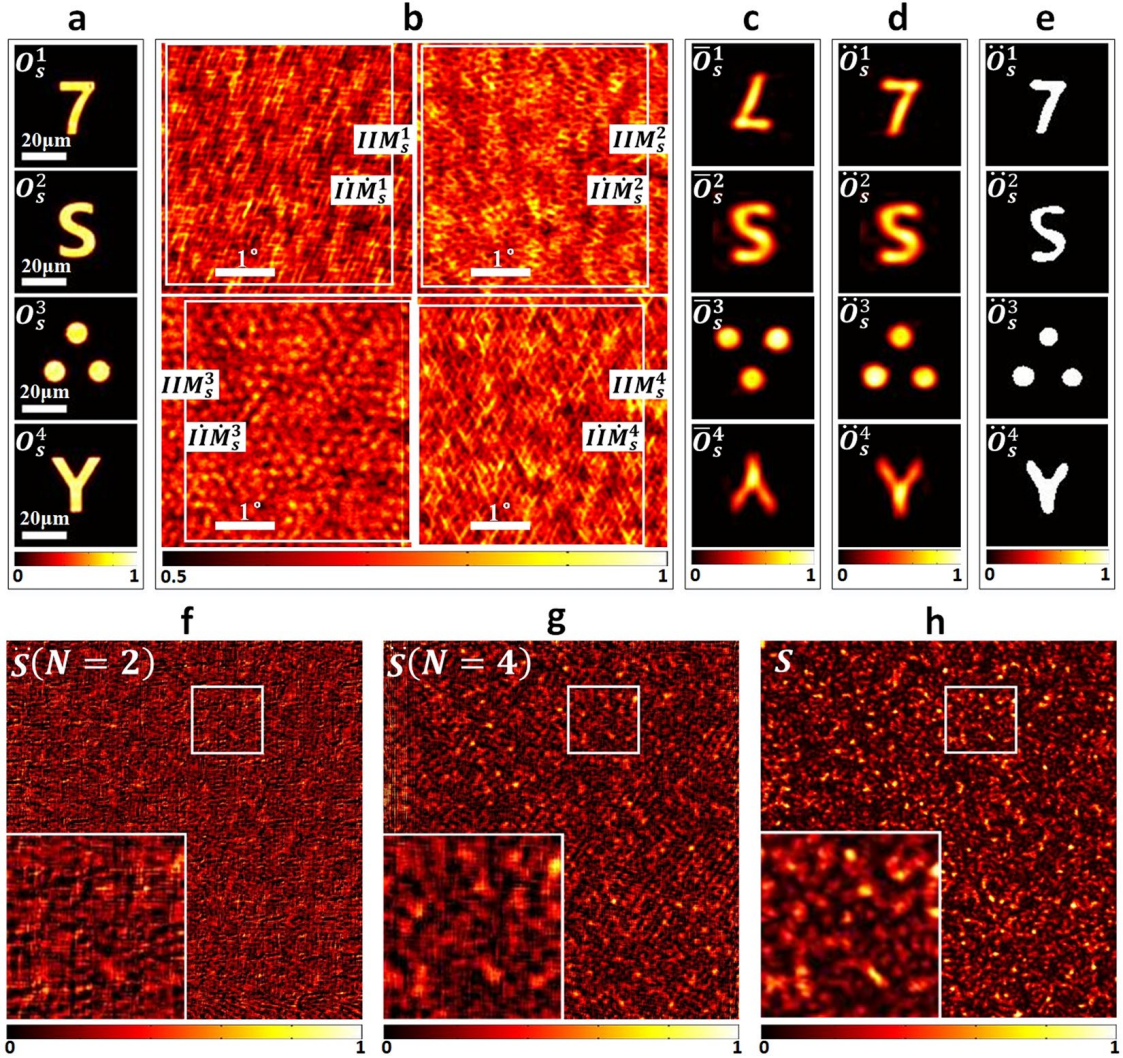
(**a**) Training targets, ${O}_{s}^{i}(i=1,2,\cdots ,4)$. (**b**) Collected IIMs of training targets, $II{M}_{s}^{i}(i=1,2,\cdots ,4)$. The regions lined in white are the aligned IIMs, $I\dot{I}{\dot{M}}_{s}^{i}(i=1,2,\cdots ,4)$. (**c**) Phase retrieval results of training targets, ${\overline{O}}_{s}^{i}(i=1,2,\cdots ,4)$. (**d**) Phase retrieval results with corrected orientations, ${\ddot{O}}_{s}^{i}(i=1,2,\cdots ,4)$. (**e**) Binarized phase retrieval results with correct orientations. The thresholds are set as 0.436, 0.592, 0.549 and 0.291, respectively. (**f**) Estimated speckle pattern, $\ddot{S}$, using two training targets (*N* = 2), ${O}_{s}^{1}$ and ${O}_{s}^{2}$. (**g**) Estimated speckle pattern, $\ddot{S}$, using the four training targets (*N* = 4). (**h**) Ground truth of speckle pattern *S*.

Four training targets, as ${O}_{s}^{1},\cdots ,{O}_{s}^{4}$ shown in Fig. 3(a), are used in the experiments. Their sizes range from 20 microns to 25 microns. The distances between the targets and scattering media, ${d}_{1}$ in Eq. (1), are set as 5 millimeters. Scanning angles are divided evenly into 600 intervals, ranging from −$2.1^\circ$ to $2.1^\circ$ in both horizontal and vertical directions. Thus, the corresponding IIMs collected are shown in Fig. 3(b) as $II{M}_{s}^{1},\cdots ,II{M}_{s}^{4}$. The reconstructed results of the training targets are shown in Fig. 3(c) as ${\overline{O}}_{s}^{1},\cdots ,{\overline{O}}_{s}^{4}$ via autocorrelation, Fourier transformation and phase-retrieval algorithm. Using the proposed cross-correlation matching method, the orientation corrected reconstruction results of the training targets are shown in Fig. 3(d) as ${\ddot{O}}_{s}^{1},\cdots ,{\ddot{O}}_{s}^{4}$ and the spatially aligned IIMs are corrupted by the white line in Fig. 3(b) as $I\dot{I}{\dot{M}}_{s}^{1},\cdots ,I\dot{I}{\dot{M}}_{s}^{4}$. Then, Eq. (15) is executed to estimate the speckle pattern by setting ${\rm{\Delta }}$ to be 0.3 and binarizing ${\ddot{O}}_{s}^{1},\cdots ,{\ddot{O}}_{s}^{4}$ to be those in Fig. 3(e) to reduce the computational complexity of solving Eq. (15). Figure 3(f) and (g) show the estimated speckle patterns using only two training targets and four training targets respectively. The ground truth of the speckle pattern is shown in Fig. 3(h). Comparing Fig. 3(f) and 3(g) with the ground truth, it can be found that both the two estimated speckle patterns are visually similar to the ground truth especially considering the low-frequency features. The result estimated using four training targets preserves more high-frequency components, which may lead to reconstructing more details of the imaging targets by deconvolution.

**Figure 4:**
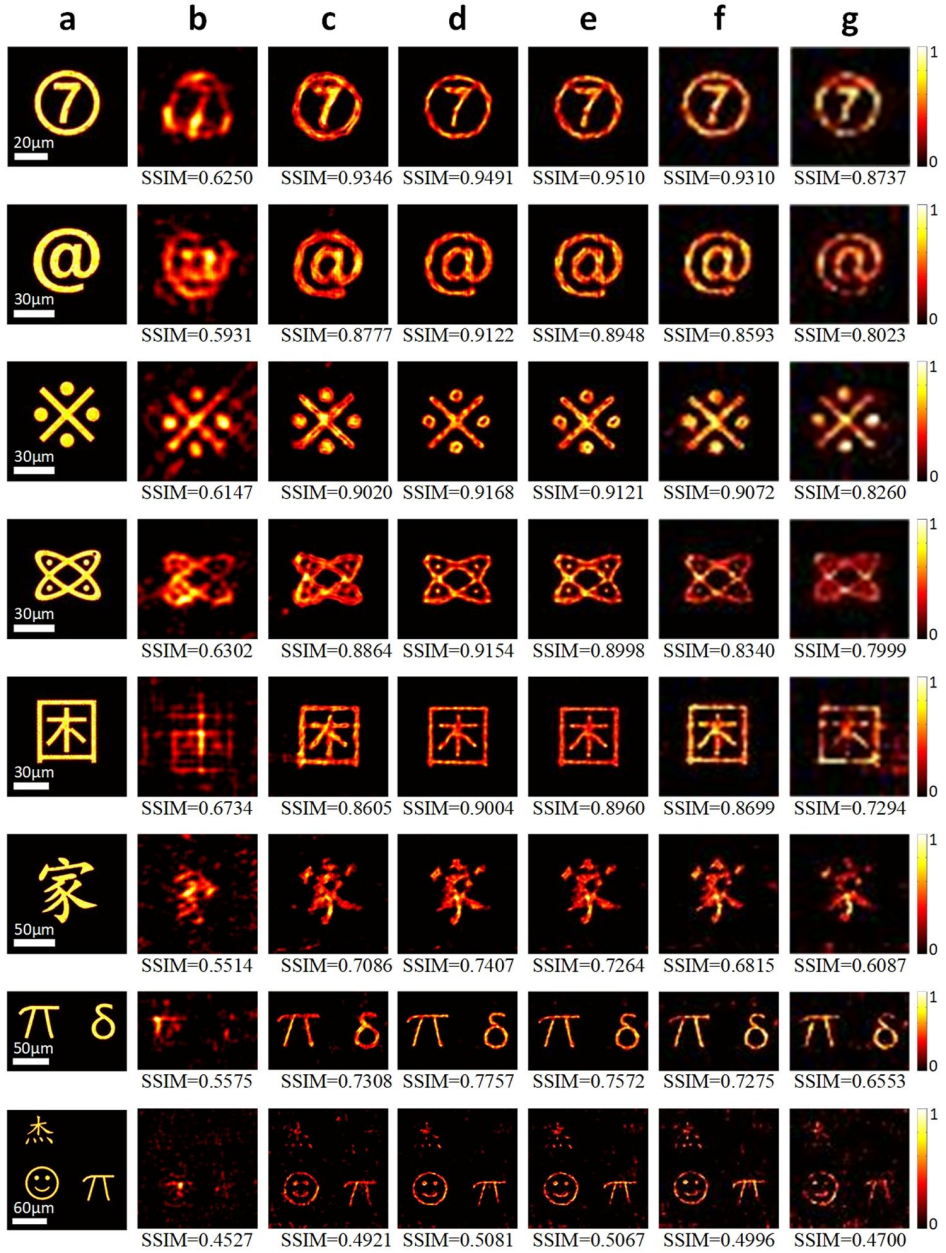
(**a**) To-be-observed targets. (**b**) Reconstructed results using phase retrieval. (**c**) Reconstructed results by deconvoluting IIMs with $\ddot{S}$ in Fig. 3(f). (**d**) Reconstructed results by deconvoluting IIMs with $\ddot{S}$ in Fig. 3(g). (**e**–**g**) Reconstructed results by deconvoluting IIMs in lower sampling rates, 1/2 × 1/2, 1/4 × 1/4 and 1/6 × 1/6, respectively, using down-sampled speckle pattern $\ddot{S}$ in Fig. 3(g). The SSIMs are calculated using images in (**a**) as ground truths.

Applying the estimated speckle pattern to the deconvolution process, the to-be-observed targets with complex structures can be reconstructed. The reconstruction quality is compared with those generated by phase retrieval^15,45,46^ to demonstrate the efficiency of the proposed method. As shown in Fig. 4(a), eight complex targets with different textural complexities and different number of subsections are tested. Their sizes range from 40 microns to 165 microns. IIMs of these targets are collected in the same way with the training targets. The reconstructed results are compared visually with the original images. Also, structural similarity index (SSIM)^55^ is calculated between the reconstructed results and the original images to evaluate the structural reconstruction capability objectively. SSIM closer to 1 corresponds to a higher reconstruction quality.

Figure 4(b) shows the reconstructed results using phase retrieval, each of which is the best result selected from performing 20 times of phase retrieval using random initial phases. It can be found that the quality of the results deteriorates sharply when targets become complex or have separated subsections. Also, because of the randomness of initial phases, the positions and shapes of the reconstructed results change a lot for each test.

Figure 4(c,d) show the reconstructed results generated by deconvoluting estimated speckle pattern in Fig. 3(f) and that in Fig. 3(g), respectively. It is obvious to see that compared with the results generated by phase retrieval, the proposed method improves the reconstruction performance for all the targets significantly, which is also robust to the structure of the targets. Although the reconstructed results derived by deconvoluting speckle pattern estimated from two training targets lose some high-frequency details, as shown in Fig. 4(c), their reconstruction qualities are still much higher than that of phase retrieval. It demonstrates that an acceptable reconstruction quality can be achieved by applying less training targets. For the targets with separated subsections, like those on the last two rows with spatial extensions more than 150 microns, the proposed method always provides reconstruction results superior to those of phase retrieval. It demonstrates a big improvement in the FoV for our method.

Figure 4(e–g) show the reconstruction results generated by deconvolution using 4, 16, 36 times lower sampling rate in collecting the IIMs of the imaging targets, as defined in Eq. (22). The speckle pattern estimated using four training targets, as shown in Fig. 3(g), is also down-sampled to be $\overline{\ddot{S}}$. The reconstructed results are up-sampled using bilinear interpolation and SSIM is calculated between the up-sampled results and the original images. It can be found that acquiring only 1/4 × 1/4 of the data, the reconstruction quality of the proposed method is still acceptable. Even if reducing the data acquired to be only 1/6 × 1/6 of those used by phase retrieval, the proposed method can still provide reconstruction quality better than that of phase retrieval. It shows high potential in significantly reducing the complexity in speckle scanning and data acquisition process for collecting IIMs, which greatly benefits the real applications.

## Conclusions

To non-invasively image complex targets through strongly scattering media with resolution beyond the limitation of illumination speckle grain lighted on the targets, speckle pattern estimation and deconvolution method is proposed. Cross-correlation matching is proposed to correct the orientations of training targets and align their corresponding IIMs, which are applied as the inputs of the speckle pattern estimation model. Both theoretical derivation and experimental results have demonstrated that the proposed speckle pattern estimation model can accurately estimate the distribution of the point-spread speckle pattern. The proposed deconvolution method is also robust even with much less data acquired. In conclusion, our imaging method relieves the complexity limitation for imaging targets, improves the reconstruction performance and reduces the data-collection complexity for the to-be-observed targets.

The principal limitation of our proposed work is the dependency on the reconstruction accuracy of training targets by phase retrieval. While, the reconstruction accuracy of phase retrieval is constrained by the relative spectral bandwidths between the imaging targets and the speckle pattern^17^. So, we put this as one of our future works to seek the way, such as introducing low rank feature of *S*, to improve the prior information for the speckle pattern estimation model, and to further analyze the solution space. Evaluating structural complexity of target from its IIM can be quite valuable in real scenario for selecting suitable training targets without phase retrieval. A preliminary experiment shows a kind of correlation between the entropy of the IIM and the phase-retrieval performance, which will be investigated further with other possible features. Another problem faced in the data collection process is the movements of imaging targets when collecting their IIMs, and it causes the structural deformation of IIMs and reconstructed results. A possible solution can be used is to track targets in real-time by analyzing the linear relationships between the variance and decorrelation time of the integrated intensity with target displacement along the axial and transversal directions, respectively^56^. Thus, the incident-angle offset of laser beam can be calculated. Beyond these, deconvoluting to-be-observed targets from different depths and introducing the deformation of memory effect can be future works as well.

## Electronic supplementary material

Supplementary Information
