# Publisher Correction: Non-invasive imaging through strongly scattering media based on speckle pattern estimation and deconvolution

**DOI:** 10.1038/s41598-018-28652-y

**Published:** 2018-07-05

**Authors:** Zhouping Wang, Xin Jin, Qionghai Dai

**Affiliations:** Graduate School at Shenzhen, Tsinghua University, Shenzhen, 518055 China

Correction to: *Scientific Reports* 10.1038/s41598-018-27467-1, published online 14 June 2018

In the HTML and PDF versions of this Article, Equation 19 is misaligned.

Equation 19 should read:

$$\begin{array}{c}A[{K}_{x}{K}_{y}\,(i-1)+\,{K}_{y}\,(m-1)+n,\,({M}_{y}+{K}_{y}-1)(m+p-2)+(n+q-1)]={\ddot{O}}_{s}^{i}(p{\rm{\Delta }}{\theta }_{x}{d}_{1},q{\rm{\Delta }}{\theta }_{y}{d}_{1}),\\ 1\,\le m\le {K}_{x},1\le n\le {K}_{y},1\le p\le {M}_{x},1\le q\le {M}_{y,}\end{array}$$

