# Supplementary material for: Non-invasive imaging through strongly scattering media based on speckle pattern estimation and deconvolution
Source: Sci Rep. 2018 Jun 14;8:9088. doi: 10.1038/s41598-018-27467-1 (PMC6002378; doi:10.1038/s41598-018-27467-1)
Supplement: Supplementary file 1 — Supplementary Information [file 41598_2018_27467_MOESM1_ESM.pdf]

Supplementary Information

Non-invasive imaging through strongly scattering media based on speckle pattern estimation and deconvolution

Zhouping Wang[1*], Xin Jin[1*] & Qionghai Dai[1]

[1]Graduate School at Shenzhen, Tsinghua University, Shenzhen, 518055, China

Corresponding author: X. Jin (E-mail: jin.xin@sz.tsinghua.edu.cn)

The speckle scanning system, which was first proposed by Bertolotti[15], is applied to the proposed work as shown in Supplementary Fig. S1(a). The light source is a 532 nm single frequency CW laser (Cobolt Samba™-100), whose diameter is 700 microns. The direction of laser beam is controlled by two galvanometric scanners in vertical and horizontal directions, respectively, to generate fixed incident point on the scattering layer with arbitrary incident angle. To simplify the calibration of the light path, transmissive imaging targets (negative film), front and back scattering layers are used in our setup. Both scattering layers are Edmund Optics 120-grit ground-glass diffusers. The distance separating the front scattering layer from the imaging targets and that separating the imaging targets and the back scattering layer are both 5 millimeters. The experiment implemented by Bertolotti[15] verifies the nearly perfect memory effect in one-degree angular range. Supplementary Fig. S1(b) is a sample image of the imaging target on the negative film captured by CCD without diffusers. The scattered image recorded by the CCD corresponding to a specific incident angle of laser beam is shown in Supplementary Fig. S1(c). The integrated intensity image corresponding to 400×400 incident angles is shown in Supplementary Fig. S1(d). The reconstructed image using phase retrieval is shown in Supplementary Fig. S1(e).

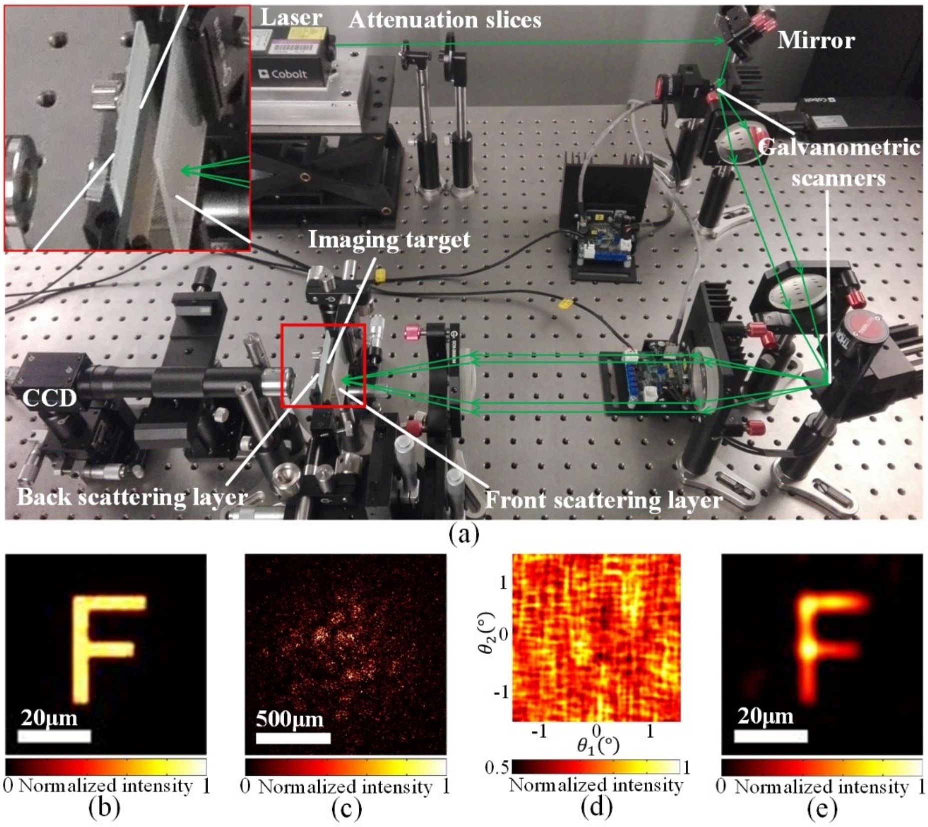

Supplementary Figure S1 | Optical setup and data collection.

(a): Optical setup; (b): Sample image of the imaging target on the negative film; (c) Scattered image captured by the CCD, which corresponds to a specific incident angle of laser beam; (d): Integrated intensity matrix, IIM, in which each pixel corresponds to the total intensity of (c) as the incident angle of laser beam is $\left(\theta_x, \theta_y\right)$; (e): Phase retrieval result with Hybrid Input-Output and Error Reduction algorithm (HIO-ER)[15,45,46].
